# Advantages of the net benefit regression framework for trial-based economic evaluations of cancer treatments: an example from the Canadian Cancer Trials Group CO.17 trial

**DOI:** 10.1186/s12885-019-5779-x

**Published:** 2019-06-07

**Authors:** Jeffrey S. Hoch, Annette Hay, Wanrudee Isaranuwatchai, Kednapa Thavorn, Natasha B. Leighl, Dongsheng Tu, Logan Trenaman, Carolyn S. Dewa, Chris O’Callaghan, Joseph Pater, Derek Jonker, Bingshu E. Chen, Nicole Mittmann

**Affiliations:** Division of Health Policy and Management, Department of Public Health Sciences and Associate Director, Center for Healthcare Policy and Research, 2103 Stockton Blvd, Sacramento, CA 95817 USA

**Keywords:** Net benefit regression, Economic evaluation, Cost-effectiveness

## Abstract

**Background:**

Economic evaluations commonly accompany trials of new treatments or interventions; however, regression methods and their corresponding advantages for the analysis of cost-effectiveness data are not widely appreciated.

**Methods:**

To illustrate regression-based economic evaluation, we review a cost-effectiveness analysis conducted by the Canadian Cancer Trials Group’s Committee on Economic Analysis and implement net benefit regression.

**Results:**

Net benefit regression offers a simple option for cost-effectiveness analyses of person-level data. By placing economic evaluation in a regression framework, regression-based techniques can facilitate the analysis and provide simple solutions to commonly encountered challenges (e.g., the need to adjust for potential confounders, identify key patient subgroups, and/or summarize “challenging” findings, like when a more effective regimen has the potential to be cost-saving).

**Conclusions:**

Economic evaluations of patient-level data (e.g., from a clinical trial) can use net benefit regression to facilitate analysis and enhance results.

## Background

We must deal with the escalating price of cancer therapy now… We cannot ignore the cumulative costs of the tests and treatments we recommend and prescribe. As the agents of change, professional societies, including their academic and practicing oncologist members, must lead the way. The time to start is now [1].

Cancer is a costly disease; there are huge costs physically, mentally and financially. A major component of many treatment regimens is pharmaceuticals. Fiscal toxicity of cancer treatment is not unique to patients and their families; healthcare payers also experience financial distress. Without the resources to pay for all treatments for all diseases for all patients, most healthcare payers have embraced an evidence informed decision-making process involving recommendation committees. Frequently, these recommendation committees embrace other types of evidence in addition to clinical evidence. For example, in Canada, the pan-Canadian Oncology Drug Review (pCODR), a national recommendation committee for oncology drugs, uses a deliberative framework that includes clinical evidence, patient values, system feasibility as well as economic evidence [2]. In the United States, the Institute for Clinical and Economic Review considers both net clinical benefit as well as value (i.e., cost-effectiveness and budget impact). Usually, the economic evidence used by recommendation committees is in the form of a cost-effectiveness model with inputs, based in part, on patient-level trial data.

In advance of formal drug reimbursement dossier submissions trial data are often presented at national conferences and published in scientific journals, providing an initial (and often impactful) preview of the clinical and economic evidence. Thus, cost-effectiveness analyses based entirely on patient-level trial data have the potential to play a major role in influencing clinical and decision maker perceptions of whether a drug provides value (e.g., is economically attractive). The analysis of a cost-effectiveness dataset provides insight into the value of the clinical benefit, over the same time horizon as the clinical study. In this way, the extra costs of the extra patient benefits accruing in the trial can be appreciated concurrently. However, there are some challenges that attend the analysis of patient-level cost-effectiveness data. For example, in cancer studies, these can involve the need to i) adjust for potential confounders, ii) identify key patient subgroups (e.g., with biomarkers), and iii) summarize the economic evidence when there is a negative cost-effectiveness ratio (e.g., when more effective treatment regimens are also potentially cost saving).

This article illustrates a regression-based method for analyzing patient-level cost-effectiveness data called net benefit regression. It has a variety of benefits that address shortcomings in conventional cost-effectiveness analysis methods. These benefits are illustrated using the Canadian Cancer Trials Group CO.17 study showing that patients with advanced colorectal cancer had improved overall survival and greater costs when cetuximab, an epidermal growth factor receptor-targeting antibody, was given in addition to best supportive care. Although the concepts of net benefit and net benefit regression have been applied in other healthcare areas, their application in oncology has not been widespread [3–5]. It is the goal of this article to clarify how to use and interpret the net benefit regression method, so that more authors and readers can appreciate what it offers.

## Methods

### Case study description

Mittmann and colleagues [6] conducted an economic evaluation of cetuximab plus best supportive care versus best supportive care alone in unselected advanced colorectal cancer patients. The initial clinical trial was conducted by the Canadian Cancer Trials Group as a multicenter, open-label, randomized phase III trial of cetuximab plus best supportive care versus best supportive care alone in patients with chemotherapy-refractory metastatic EGFR-positive colorectal cancer (ClinicalTrials.gov number NCT00079066). Survival times for the entire study population and for patients whose tumors harbored wild-type *KRAS* were calculated over an 18- to 19-month period [6], and the trial (hereafter referred to as CO.17) found a statistically significant overall survival advantage for cetuximab with a 1.5 month difference in median survival for cetuximab versus best supportive care [7]. In patients with wild-type *KRAS* tumors, there was a larger survival advantage (i.e., 4.7 months additional median survival for cetuximab) [8].

Mittmann and colleagues conducted a cost-effectiveness analysis using prospectively collected cost and quality adjusted life year (QALY) data for patients in the CO.17 [6]. For patients in the trial, cetuximab showed unattractively high incremental cost-effectiveness ratios. The incremental cost-effectiveness ratios (ICERs) were more favorable for patients whose tumors harbored wild-type *KRAS* but were still more than $186,000 per quality-adjusted life-year gained. Since there is no universally agreed upon cost-effectiveness threshold or willingness to pay (WTP) value, jurisdictions often adopt fuzzy thresholds that are guided by several factors [9–11]. Nevertheless, the likelihood of a positive funding recommendation appears inversely related to the incremental cost-effectiveness ratio (i.e., higher ICERs have a lower probability of being funded). [12, 13] This suggest that cost-effectiveness methods that explicitly allow the WTP threshold to vary may be helpful.

In the following section, we describe net benefit regression before applying the technique to analyze the cost-effectiveness data for patients in the CO.17 study.

### Net benefit regression framework

We briefly review below the key components of net benefit regression and offer additional references for the interested reader [14–16]. With the net benefit regression approach, analysts can use regression-based techniques to analyze cost-effectiveness data; some advantages of the net benefit regression approach include facilitating solutions to challenging statistical situations (e.g., negative cost-effectiveness ratios or when Fieller’s theorem will not yield a confidence interval) [14]. The net benefit regression framework was proposed a decade ago to marry regression and cost-effectiveness methods [17]. At that time, the conventional statistic reported in most cost-effectiveness studies was the ICER.

### Building from the ICER

Mathematically, the ICER estimate is defined as Extra Cost ÷ Extra Effect, where Extra Cost is defined as ΔC = Expected Cost with New Treatment - Expected Cost with Usual Care and Extra Effect is defined as ΔE = Expected Effect with New Treatment - Expected Effect with Usual Care. With a cost-effectiveness dataset, it is common to use the Average Cost and Average Effect to represent Expected values. The ICER is troublesome to estimate because it is a ratio; however, its parts—the numerator and denominator—can be estimated easily by regression.

If one defines a binary treatment indicator variable as TX = 1 for a study participant receiving the new treatment, and TX = 0 for a study participant receiving usual care, then one can use ordinary least squares (OLS) to estimate linear regressions for cost (c_i_) and effect (e_i_). By adding an interaction term (say, between the *KRAS* status and TX indicator variables), it is possible to explore hypothesis-generating questions about subgroups for whom the new intervention may be more (or less) cost-effective. For example, is a drug more cost-effective for patients with wild-type *KRAS* tumors?

### Willingness to pay (WTP)

When a new treatment costs more (ΔC > 0) and is more effective (ΔE > 0), the ICER > 0. For decisions, an ICER must be compared with a WTP threshold value. Unfortunately, a decision maker’s WTP is unknown, so methods that treat WTP as unknown are best (e.g., varying WTP and exploring how a recommendation based on the estimated ICER may change). Net benefit regression addresses the unknown nature of the “correct” WTP value within the incremental net benefit.

### Incremental net benefit regression

By computing each patient’s net benefit (NB) as WTP × e_i_ − c_i_ and using it for a dependent variable, one can run a simple or multiple linear regression of the form

or $$ \mathrm{NB}={\mathrm{b}}_0+{\mathrm{b}}_{\mathrm{TX}}\mathrm{TX}+{\upvarepsilon}_{\mathrm{NB}} $$

respectively $$ \mathrm{NB}={\mathrm{b}}_0+{\mathrm{b}}_{\mathrm{TX}}\mathrm{TX}+{\mathrm{b}}_1{\mathrm{X}}_1+\cdots +{\mathrm{b}}_{\mathrm{p}}{\mathrm{X}}_{\mathrm{p}}+{\upvarepsilon}_{\mathrm{NB}}, $$

If b_TX_ > 0, the new treatment is cost-effective since b_TX_ equals the incremental net benefit (INB); the INB conveys by how much the value of the extra effect outweighs the extra cost (i.e., INB = WTP × ΔE − ΔC) [17]. Another way to view the INB is as the difference in the average net benefits between the new treatment and usual care: new treatment is more cost-effective if it has higher net benefits than usual care. The linearity of the dependent variable NB means the estimate of b_TX_ = WTP × ΔE − ΔC. While the 95% confidence interval (CI) for the ICER cannot be made from the separate CIs for the estimates of ΔC and ΔE (because this process ignores the correlation between the cost and effect data) [18], the 95% CI for b_TX_ is the 95% CI for the INB. If there is concern about using a parametric method for the 95% CI, one can use a non-parametric method like bootstrapping [19].

By estimating net benefit regression equations with various WTP values, one can gauge the sensitivity of cost-effectiveness findings in relation to WTP assumptions. One WTP value that should always be checked in a net benefit regression is WTP = $ΔC/ΔE since this should yield an INB estimate of zero (i.e., b_TX_ = 0). By setting WTP = $0, the INB should become − 1 × ΔC. One can characterize uncertainty using CIs or *p*-values to create cost-effectiveness acceptability curves (e.g., see [20] for a step by step tutorial on using *p*-values this way). Because the INB and the ICER are related through WTP, both their estimates and uncertainty are closely connected. A graph of INB by WTP has a y-intercept equal to -ΔC, a slope of ΔE and an x-intercept of the ICER. The addition to the graph of 95% CIs for the INB illustrates, at their x-intercepts, the lower and upper 95% CIs (from Fieller’s Theorem) for the ICER (see Results section for examples). We illustrate these points next using net benefit regression results.

## Results

Table 1 reports the results of simple linear regressions with dependent variables Effect, Cost and NB regressed on the cetuximab treatment indicator (i.e., the TX variable in the **METHODS** section). The estimates in Table 1 represent ΔE, ΔC and ΔNB (i.e., INB), respectively. Results when WTP = $0 are reported in the NB ($0) column; results for WTP = $500,000 are reported in the NB ($500 k) column. In this economic analysis, cetuximab showed extra cost of $22,210 and extra effect of 0.0771 QALYs (see the row labeled “ALL” in Table 1) when compared with best supportive care for all patients in the CO.17 trial. This corresponds to an ICER over $288,000 (i.e., 22,210/0.0771), not generally considered economically attractive. However, the results differ by *KRAS* status. While the extra cost estimate appears larger for patients with wild-type *KRAS* tumors (ΔC = $30,843, *p*-value < 0.001) than patients whose tumors do not express wild-type *KRAS* (ΔC = $13,787, *p*-value < 0.001), the extra effect estimates tell a much different story. Cetuximab appears more effective than best supportive care for patients with wild-type *KRAS* tumors (ΔE = 0.1769 QALYs, *p*-value < 0.001) but less effective than best supportive care for patients whose tumors do not express wild-type *KRAS* (ΔE = − 0.0172 QALYs, *p*-value > 0.40).

**Table 1:** Simple linear regression estimates producing estimates of incremental values (i.e., Δ’s)

Regression Subgroup	Simple Linear Regression Estimates of Incremental Net Benefit (*p*-values)								
	Effect (in QALYs)	Cost (in $)	NB($0) (amount by which benefits outweigh costs, when an extra QALY is worth $0)	NB($50 k) (amount by which benefits outweigh costs, when an extra QALY is worth $50,000)	NB($100 k) (amount by which benefits outweigh costs, when an extra QALY is worth $100,000)	NB($200 k) (amount by which benefits outweigh costs, when an extra QALY is worth $200,000)	NB($300 k) (amount by which benefits outweigh costs, when an extra QALY is worth $300,000)	NB($400 k) (amount by which benefits outweigh costs, when an extra QALY is worth $400,000)	NB($500 k) (amount by which benefits outweigh costs, when an extra QALY is worth $500,000)
KRAS-MUT (*n* = 150)	−0.0172 (0.4158)	13,787 (< 0.001)	− 13,787 (< 0.0001)	− 14,650 (< 0.0001)	−15,514 (0.0007)	−17,240 (0.0573)	−18,966 (0.1664)	−20,692 (0.2601)	−22,418 (0.3308)
KRAS-WT (*n* = 216)	0.1769 (< 0.001)	30,843 (< 0.001)	−30,843 (< 0.0001)	−21,999 (< 0.0001)	− 13,154 (0.0013)	4536 (0.5734)	22,226 (0.0715)	39,916 (0.0167)	57,606 (0.0062)
ALL (*n* = 527)	0.0771 (0.0011)	22,210 (< 0.001)	−22,210 (< 0.0001)	−18,358 (< 0.0001)	−14,507 (< 0.0001)	−6805 (0.1789)	898 (0.9080)	8600 (0.4129)	16,303 (0.2186)

Note: The *p*-value is reported in parenthesis below the coefficient estimate. This two-sided *p*-value is converted to a 1-sided *p*-value [20] to make the cost-effectiveness acceptability curve (CEAC) in Figure 2

Table 2 shows the estimates for multiple linear regression; these results further support analyzing the data stratified by *KRAS* status. The coefficient on the cetuximab treatment indicator, which represents INB for patients with mutant status, is negative for WTP values from $0 to $500,000. However, the interaction term between cetuximab treatment and wild-type *KRAS* status, which represents difference of INBs between patients with *KRAS* wild-type and mutant statuses, switches from a negative value (− 738) to a positive value (14,762) as WTP increases from $100,000 to $200,000. This coincides with the INB estimate transitioning from a negative value (− 14,637) to a positive value (1149) over the same WTP range for patients whose tumors express wild-type *KRAS* (*KRAS-WT*). For the lowest value of WTP ($0), the interaction term between the cetuximab treatment and *KRAS*-WT indicator variables is statistically significantly negative (− 16,238, *p*-value < 0.001). This implies that ΔC is significantly higher for patients with wild-type *KRAS* than those with mutant *KRAS*. Conversely, for the highest value of WTP ($500,000), the interaction term is statistically significantly positive (61,262, *p*-value < 0.05); this suggests INB with this WTP is significantly higher for patients with wild-type *KRAS* than those with mutant *KRAS*.

**Table 2:** Multiple linear regression with Willingness to Pay (WTP) ranging from $0 to $500,000

Regression Key covariates	Multiple Linear Regression Estimates of Incremental Net Benefit [SE] (p-value)						
	NB($0) (amount by which benefits outweigh costs, when an extra QALY is worth $0)	NB($50 k) (amount by which benefits outweigh costs, when an extra QALY is worth $50,000)	NB($100 k) (amount by which benefits outweigh costs, when an extra QALY is worth $100,000)	NB($200 k) (amount by which benefits outweigh costs, when an extra QALY is worth $200,000)	NB($300 k) (amount by which benefits outweigh costs, when an extra QALY is worth $300,000)	NB($400 k) (amount by which benefits outweigh costs, when an extra QALY is worth $400,000)	NB($500 k) (amount by which benefits outweigh costs, when an extra QALY is worth $500,000)
cetuximab TX indicator	−14,185 [2409] (< 0.0001)	−14,042 [2667] (< 0.0001)	− 13,899 [4403] (0.0017)	− 13,612 [8766] (0.1214)	− 13,326 [13,348] (0.3188)	− 13,040 [17,982] (0.4688)	−12,754 [22,637] (0.5735)
KRAS-WT	1210 [2289] (0.5975)	2458 [2534] (0.3327)	3707 [4184] (0.3763)	6204 [8329] (0.4569)	8701 [12,683] (0.4932)	11,198 [17,086] (0.5127)	13,695 [21,509] (0.5248)
cetuximab TX × KRAS-WT	− 16,238 [3165] (< 0.0001)	− 8488 [3505] (0.0160)	−738 [5787] (0.8986)	14,762 [11,520] (0.2009)	30,262 [17,541] (0.0854)	45,762 [23,631] (0.0536)	61,262 [29,747] (0.0402)
Overall INB estimate of cetuximab for KRAS-WT patients, $	−30,422	−22,530	− 14,637	1149	16,935	32,721	48,507

Note: The reported regression coefficients were estimated while adjusting for Age, Sex, ECOG performance status, Site of primary cancer (e.g., colon only, rectum only or colon and rectum), Any previous radiotherapy, Previous chemotherapy variables (e.g., Adjuvant therapy, Number of regimens, Irinotecan, Oxaliplatin, Thymidylate synthase inhibitor), Site of disease, Number of sites of disease. The *p*-value is reported in parenthesis below the standard error in brackets

**Fig. 1:**
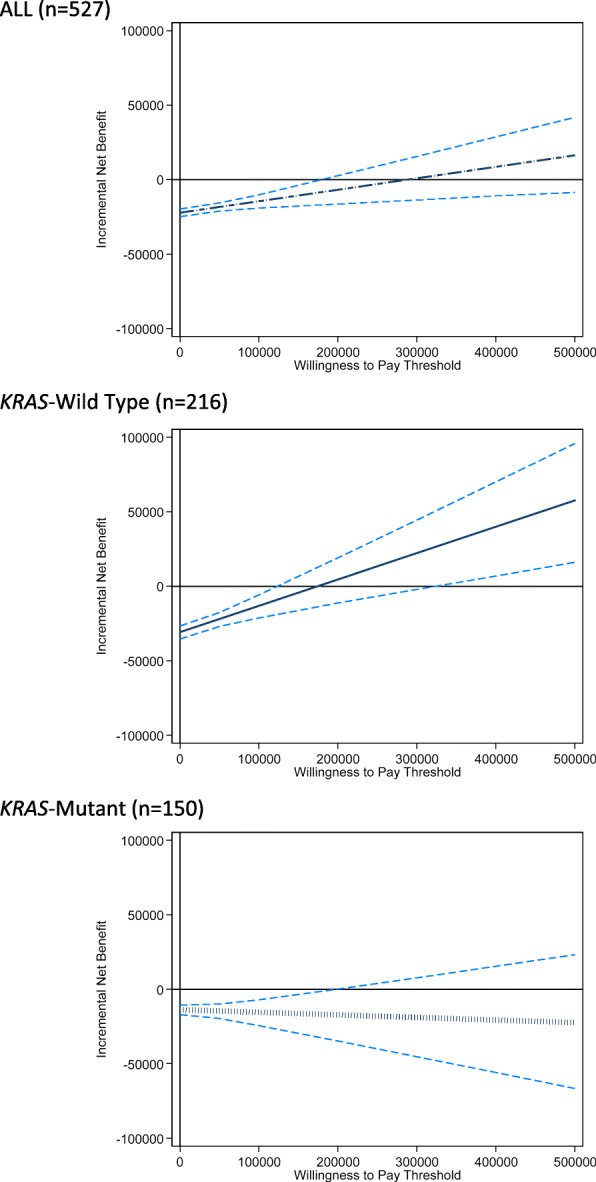
Incremental net benefit estimate for all patients (upper graph, dashed line), KRAS-WT (middle graph, solid line), and KRAS-MUT (lower graph, hashed line) and 95% confidence intervals (dashed lines)

**Fig. 2:**
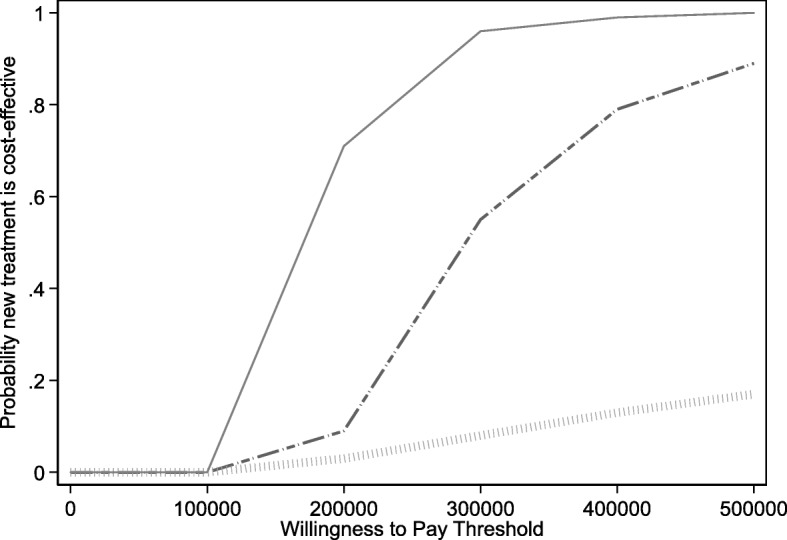
Probability that new treatment is cost-effective for KRAS-WT (upper solid line), all patients (middle dashed line) and KRAS-MUT (lower hashed line) by Willingness to Pay threshold values

Figure 1 plots the incremental net benefit estimate (as a solid line) and the pointwise 95% CIs (as dashed lines) in relation to WTP values varying from $0 to $500,000. Vertical values greater than zero indicate when INB is positive and cetuximab is cost-effective. The graphs for the overall sample and the *KRAS*-WT sub-group (the top and middle graphs in Fig. 1) show a positively sloped INB line that intersects the horizontal axis; for the overall sample this occurs near the WTP value of $300,000 and near $200,000 for the *KRAS-WT* subgroup. For the patients whose tumors do not express wild-type *KRAS* (i.e., the *KRAS*-MUT group), the negatively sloped INB line does not intersect any positive WTP value (on the horizontal axis). Figure 2 communicates the probability that cetuximab is cost-effective as WTP varies. There are three curves: one for *KRAS*-WT patients (upper solid line), one for all patients (middle dashed line) and one for *KRAS*-MUT patients (lower hashed line).

## Discussion

Typically, ICERs are the metrics reported in economic evaluations; However, in this case study, the ICER for the patients whose tumors do not express wild-type *KRAS* (*KRAS*-MUT) is negative. Based on expert recommendations, this means the ICER should not be calculated [21]. This makes it challenging to report the conventional cost-effectiveness statistic (which is negative in this case) and to report its 95% CI (where at least one limit will be negative as well). In contrast, Table 1’s negative INB estimates, reported for all WTP values, indicate that cetuximab for *KRAS*-MUT patients is not economically attractive (at least for WTP values from $0 to $500,000). For *KRAS*-WT patients, the INB estimate becomes positive (switching from − 13,154 to 4536) as WTP increases from $100,000 to $200,000. This indicates that the ICER falls within this range (ΔC/ΔE = 30,843/0.1769 ≈ 174,350 per QALY). The overall sample demonstrates a similar pattern, switching from − 6805 to 898 as the WTP increases from $200,000 to $300,000 due to the overall ICER being ΔC/ΔE = 22,210/0.0771 ≈ $288,000 per QALY. As noted earlier, when WTP = $0, the INB estimate reduces to −ΔC; this explains the similarity between the coefficients in the Cost Column and those in the NB($0) column in Table 1.

As noted earlier, the findings in Table 2 support stratifying the analysis by *KRAS* status. Either simple linear or multiple linear regression can be run separately stratifying on a patient’s tumor’s *KRAS* status. In this case study, we simplified matters by focusing on simple linear regressions (except for Table 2). The findings of the simple linear regression models were similar to those of the multiple linear regression models for small WTP values; they diverged more for larger WTP values. This suggests that there is important variability in the patient outcome related to the independent variables; however, the variability in cost is not as strongly associated with the independent variables since adjusting for the patient covariates (i.e., all of the X_p_’s) does not affect the INB estimate for small WTP values. In passing, we note that investigators interested in studying a patient subgroup, defined by a continuous variable (e.g., age, disease severity, etc.), would not be able to stratify and run separate models; a model with a treatment interaction term would be better suited to exploring this type of hypothesis generating question (involving a continuous covariate).

Figure 1 demonstrates the usefulness of an INB by WTP graph. The different shapes of the curves suggest different findings. The upper graph (for the overall sample) and the middle graph (for the *KRAS*-WT group) show INB lines with negative y-intercepts, positive slopes and x-intercepts at WTP values of approximately $300,000 (for the overall sample) and approximately $200,000 (for the *KRAS-WT* group). As noted in the Methods section, an INB by WTP graph has a y-intercept equal to -ΔC, a slope of ΔE and an x-intercept of the ICER. Thus, a negative y-intercept means cetuximab is more costly, a positive slope means that cetuximab is more effective, and the WTP value where the INB estimate line intersects is the ICER. Of the three graphs in Fig. 1, the KRAS-WT group has the steepest INB estimate line; therefore, that group enjoys the largest gain from treatment (i.e., has the biggest ΔE). For the KRAS-MUT group, the negative y-intercept means cetuximab is more costly, the slightly negative slope means that cetuximab is slightly *less* effective than best supportive care, and the WTP value where the INB estimate line looks to intersect indicates a negative ICER.

Figure 1 can also be used to characterize the uncertainty associated with both the INB *and* the ICER. For the *KRAS*-WT group (in the middle graph), the upper and lower 95% confidence limits *for the INB* intersect the horizontal axis over the illustrated WTP range of $0 to $500,000. The two intersection points mark the Fieller’s Theorem 95% CI *for the ICER*. This 95% CI corresponds very closely to the 95% CI of $130,326 to 334,940 reported in the original economic analysis. For the overall sample, Mittmann and colleagues reported a 95% CI of $187,440 to 898,201. This is congruent with the upper graph in Fig. 1; one confidence limit intersects the horizontal axis near a WTP = $200,000 and the other intersection point appears greater than $500,000. The lower graph (for the *KRAS*-MUT group) suggests a negative ICER with one 95% confidence limit that will be negative.

The cost-effectiveness acceptability curve (CEAC) in Fig. 2 combines parts of the regression results and Fig. 1 to characterize uncertainty [20]. The CEAC shows the probability that cetuximab plus best supportive care is cost-effective compared to best supportive care alone. WTP varies along the horizontal axis reflecting its unknown nature (to the analyst). The vertical axis communicates the portion of the INB distribution that is positive (indicating the probability that cetuximab is cost-effective). The three curves—one for *KRAS*-WT patients (upper solid line), one for all patients (middle dashed line) and one for *KRAS*-MUT patients (lower solid line)—support the general conclusions that have been offered. While the CEACs presented in Fig. 2 were made using parametric *p*-values from Table 1, it is possible to create them using non-parametric bootstrapping methods [20].

### Limitations

We conclude our discussion by reviewing some key limitations in our example involving the analysis of person-level cost-effectiveness data. The usefulness of person-level cost-effectiveness data is diminished when either a relevant outcome is not included in the original study or when the trial is too short in duration to see activity in the outcome of interest. The original clinical trial in our example used overall survival as its primary end point with secondary outcomes that included progression-free survival as well as quality adjusted life years (QALYs). Even with the strength of the trial’s design, there is still the critical question of whether “enough” study participants contributed outcome data. Of the randomly assigned 572 patients, a total of 456 deaths occurred by the date of analysis. The median survival was 6.1 months in the cetuximab group and 4.6 months in the supportive-care group. The proportions of patients surviving at 6 and 12 months were 50 and 21%, respectively, in the cetuximab group and 33 and 16%, respectively, in the supportive care group.

Typically, when time-to-event data (e.g., survival) are incomplete, methods for censored data are employed. In contrast to the original economic evaluation which employed two methods to calculate overall survival: the restricted mean survival method (which restricts calculation of mean survival to the longest observed survival time) and the Kaplan – Meier method (which takes into account censoring), we used only the restricted mean survival method. Our simplifications (e.g., ordinary least squares to estimate a simple linear regression without specific methods for censoring) did not appear to make any qualitative difference in this case study; the original economic evaluation reported ICERs of $186,761 (*KRAS*-WT) and $299,613 (entire study population) compared to ICERs of $174,353 and $288,067 calculated using estimates from our Table 1, respectively. While our simple illustration of net benefit regression is meant to facilitate understanding, there are situations where more advanced methods for the analysis of censored cost-effectiveness data may be desired. Advanced papers by Bang and Tsiatis [22] as well as Chen et al. [23] provide excellent direction in this area. More advanced methods for simultaneous estimation of cost and effect equations are also available [24].

Finally, often analysts do not report INB results for *all* willingness to pay (WTP) values. When a reader is interested in a WTP value occurring within the range of WTP values that are used (e.g., WTP = $123,456) or a WTP value outside the range (e.g. WTP = $600,000), this may appear to be a concern. This concern can be addressed easily because the formula for INB is linear (i.e., INB = WTP × ΔE − ΔC). For each $1 change in WTP, the INB changes by ΔE. Thus, the INB when WTP = $123,456 is $23,456 × ΔE more than the INB when WTP = $100,000. Using Table 1 and *KRAS*-WT as an example, INB($123,456) = INB($100,000) + ($23,456 × ΔE) = − 13,154 + ($23,456 × 0.1769) = −$9005. This matches the result from a direct calculation of INB($123,456) = $123,456 × ΔE − ΔC = $123,456 × 0.1769 − $30,843 ≈ −$9005. This method can also be used for WTP values outside of the WTP ranges reported. For example, using the values in Table 1 for *KRAS*-WT, INB($600,000) = INB($500,000) + ($100,000 × ΔE) = 57,606 + ($100,000 × 0.1769) = $75,296.

Direct calculation verifies this result for *KRAS*-WT, INB($600,000) = $600,000 × 0.1769 − $30,843 ≈ $75,297.

## Conclusion

This article showcases the advantages of the net benefit regression framework [17]. The framework allows incremental cost and incremental effect to be estimated either separately (i.e., using cost or effect as a dependent variable) or together (i.e., using net benefit as a dependent variable). In this paper’s case study, there was a straightforward application of OLS. However, more ambitious analytical strategies with more sophisticated techniques can be used (e.g., using regression diagnostics, employing interaction terms and/or using advanced methods for non-randomized data). We were able to adjust our cost-effectiveness analysis for covariates using multiple linear regression and to explore clinically relevant patient subgroups. The incremental net benefit by WTP curve illustrated both our estimate of cost-effectiveness and the associated uncertainty. The INB by WTP graph allows the cost-effectiveness results to reflect the unknown WTP’s impact on policy implications. When analyzing a cost-effectiveness dataset, net benefit regression can be a useful starting point for exploring one’s data and communicating a new treatment’s value.

## Data Availability

The datasets analysed during the current study are not publicly available due to the Canadian Cancer Trials Group’s policy. However, the results can verified based on this analysis and the separate publication of the original cost-effectiveness article.

## Abbreviations

CEAC: Cost-effectiveness acceptability curve
CI: Confidence interval
ICER: Incremental cost-effectiveness ratio
INB: Incremental net benefit
MLR: Multiple linear regression
MUT: Mutant
NB: Net benefit
OLS: Ordinary least squares
QALY: Quality-adjusted life-year
SLR: Simple linear regression
WT: Wild type
WTP: Willingness to pay
